# Troxerutin Reduces Kidney Damage against BDE-47-Induced Apoptosis via Inhibiting NOX2 Activity and Increasing Nrf2 Activity

**DOI:** 10.1155/2017/6034692

**Published:** 2017-10-15

**Authors:** Qun Shan, Juan Zhuang, Guihong Zheng, Zifeng Zhang, Yanqiu Zhang, Jun Lu, Yuanlin Zheng

**Affiliations:** ^1^School of Environment Science and Spatial Informatics, China University of Mining and Technology, Xuzhou, Jiangsu Province 221008, China; ^2^Key Laboratory for Biotechnology on Medicinal Plants of Jiangsu Province, School of Life Science, Jiangsu Normal University, 101 Shanghai Road, Xuzhou, Jiangsu Province 221116, China

## Abstract

2,2,4,4-Tetrabromodiphenyl ether (BDE-47), one of the persistent organic pollutants, seriously influences the quality of life; however, its pathological mechanism remains unclear. Troxerutin is a flavonoid with pharmacological activity of antioxidation and anti-inflammation. In the present study, we investigated troxerutin against BDE-47-induced kidney cell apoptosis and explored the underlying mechanism. The results show that troxerutin reduced renal cell apoptosis and urinary protein secretion in BDE-47-treated mice. Western blot analysis shows that troxerutin supplement enhanced the ratio of Bcl-2/Bax; inhibited the release of cytochrome c from mitochondria, the activation of procaspase-9 and procaspase-3, and the cleavage of PARP; and reduced FAS, FASL, and caspase-8 levels induced by BDE-47. In addition, troxerutin decreased the production of reactive oxygen species (ROS) and increased the activities of antioxidative enzymes. Furthermore, troxerutin blunted Nrf2 ubiquitylation, enhanced the activity of Nrf2, decreased the activity of NOX2, and ameliorated kidney oxidant status of BDE-47-treated mice. Together, these results confirm that troxerutin could alleviate the cytotoxicity of BDE-47 through antioxidation and antiapoptosis, which suggests that its protective mechanism is involved in the inhibition of apoptosis via suppressing NOX2 activity and increasing Nrf2 signaling pathway.

## 1. Introduction

Polybrominated diphenyl ethers (PBDEs), a kind of persistent organic pollutants (POPs), have been emerging in tremendous danger on the global environment and human health. How to prevent and treat the damage of PBDEs has become a close attention. To find specific effective techniques and drugs against PBDEs in treatment, to a large extent, depends on the molecular mechanism underlying the effect of PBDEs on tissue damage. Among 209 homologues, 2,2,4,4-tetra-brominated diphenyl ether (BDE-47) is widely distributed in biological materials with high contents. Seriously, accumulation of BDE-47 in the body causes the severe damage of tissues including the liver, kidney, and adipose tissue [1–11]. For example, the homeostasis of parathyroid hormone is disturbed by BDE-47 via decreasing the concentration of triiodothyronine (T3) and tetraiodothyronine (T4) in the blood serum of human or mouse [11]. Furthermore, the neurotoxicity of BDE-47 leads to a disordered behavior because of the decrease of synaptic protein in the central nervous system of mice [5–7, 9]. Recent findings show that BDE-47 induces apoptosis of human embryonic kidney cells, resulting in metabolic dysfunction [12]. However, the toxicological effects and underlying mechanism of kidney damage induced by BDE-47 in vivo are unclear.

Further studies show that natural products *α*-tocopherol, omega-3 polyunsaturated fatty acids, and troxerutin may be efficient candidate compounds against the damage induced by BDE-47 [13–16]. Troxerutin is a derivative of naturally occurring bioflavonoid rutin and enriched in fruits, vegetables, tea, coffee, and cereals. Due to its good solubility in water, troxerutin is easily absorbed by the gastrointestinal system and exhibits effectively protective function without cytotoxicity on tissues. Clinically, troxerutin has been available in the treatment of phlebocholosis and hemorrhoidal disease because it dredges the blood vessel well, improves the microcirculation, and protects the endothelial cells [17–19]; in addition, troxerutin possesses potential efficacy in the treatment of diabetic mellitus and Alzheimer's disease partly via its antioxidant activity [20, 21]. Recently, we find that troxerutin significantly reduced the damage of several tissues including the brain, liver, and kidney through ameliorating antioxidant level in D-galactose-induced aging mice [22–24] and, consequently, improved memory deficits via suppressing inflammatory response and oxidative stress [25, 26]. Troxerutin enhanced the metabolism ability in lipid in high-fat diet-treated mice [27] and protected against liver damage caused by BDE-47 [15]. In the present study, we investigate the functional protective role of troxerutin in the kidney and the mechanism underlying the impairment caused by BDE-47 in a C57BL/6J mouse model.

## 2. Materials and Methods

### 2.1. Animal and Treatment

The dose of BDE-47 (50 mg/kg/day) was based on the previous data [15] and our preliminary experiments (seen in Supplement materials available online at https://doi.org/10.1155/2017/6034692). Male C57BL/6J mice (6 weeks) were obtained from the Branch of National Breeder Center of Rodents (Beijing Vital River Laboratory Animal Technology Co. Ltd., Beijing, China). The mice were kept under the conditions of constant temperature (22–24°C) and humidity (60%) and a 12 h light/dark schedule and given free access to normal food and water. After one week of acclimatization to the conditions, mice were randomly divided into four groups including control group, BDE-47-treated group, BDE-47/troxerutin-cotreated group, and troxerutin-treated group. BDE47-treated mice were administrated orally with BDE-47 at a dose of 50 mg/kg/d [>98% purity, Chem Service, West Chester, PA, USA] or its solvent (corn oil) for 12 weeks. Troxerutin treatment was as follows: four hours after BDE-47 administration, the mice of BDE-47 + troxerutin and troxerutin groups obtained 100 mg/kg/day troxerutin (dissolved in distilled water containing 0.1% Tween 80; >99% purity, Baoji Fangsheng Biotechnology Co. Ltd., Baoji, China) by gavage, and the mice of the control and BDE-47 groups were given equal solvent. All procedures in the experiment were conformed to the legislation on the use and care of laboratory animals and were approved by the respective university committee for animal experiments. After 12 weeks, mice were sacrificed and kidney tissues were used for experiments or stored at −80°C for later use.

### 2.2. Urine Collection and Determination of Albumin and Creatinine

Urine samples were collected from the mice housed in metabolic cages for 24 h. The excretion of urine protein was evaluated using urine albumin-to-creatinine ratio (ACR) in 24 h urine collections. The content of urine creatinine and albumin was measured using the commercial kits (Jiancheng Institute of Biotechnology, Nanjing, China). The absorbed value was examined by ultraviolet/visible spectrometer (UV-2501PC, Shimadzu, Japan).

### 2.3. Determination of Redox Status

#### 2.3.1. ROS Assay

Reactive oxygen species (ROS) was measured as OxiSelect™ In Vitro ROS/RNS Assay Kit (Cell Biolabs Inc., San Diego, CA, USA). In brief, mice were deeply anaesthetized and sacrificed. The kidney tissues were immediately separated, homogenized, and sonicated in ice cold 1/20 (*w*/*v*) 50 mM phosphate buffer saline solution (PBS, pH 7.2). Homogenates were centrifuged at 10000*g* for 5 min to obtain the supernatants for detecting the level of ROS. 50 *μ*l (*V*_homogenate: PBS_ = 1 : 4) homogenate samples were added to wells of a 96-well plate for fluorescence assay and then added with 50 *μ*l of catalyst to each well, mixed well, and incubated 5 minutes at room temperature. 100 *μ*l of fluorescent probe 2,7-dichlorofluorescein diacetate (DCFH-DA) was added to each well and incubated at room temperature under dark conditions. After 30 min of incubation, the conversion of DCFH-DA to the fluorescent product DCF was measured using a spectrofluorometer with excitation at 484 nm and emission at 530 nm. Blanks were included to correct for background fluorescence (conversion of DCFH-DA in the absence of homogenate). ROS formation was quantified from a DCF standard curve. Data are expressed as nmol of DCF formed per minute per mg of protein.

#### 2.3.2. The Determination of Antioxidant Indexes of Kidney Tissue

The renal tissues were taken out and homogenized in 1 : 5 (*w*/*v*) pH 7.2 PBS buffer with 10 strokes at 1200 rpm in a Potter homogenizer at 4°C. The homogenates were centrifuged at 3000 rpm for 15 min at 4°C, and the supernatants were collected for the detection of the following indices: glutathione (GSH), superoxide dismutase (SOD), catalase (CAT), and glutathione peroxidase (GPx). The experimental procedures were strictly carried out according to the kit instructions (Nanjing Jiancheng Bioengineering Institute, Nanjing, China). GSH content, GPx activity, and SOD activity were expressed as U/mg protein, and CAT activity was showed as nM H_2_O_2_ decomposed/min/mg protein.

### 2.4. Apoptosis Detection

Terminal deoxynucleotidyl transferase-mediated dUTP nick end labeling (TUNEL) assay was performed on 4% paraformaldehyde (PFA) fixed sections of the kidney tissue and was carried out according to the instruction of in situ cell death detection kit (Roche Biomedical Laboratories Inc., Burlington, Germany). The protocol is as follows: the sections were fixed in 4% PFA in pH 7.2 PBS at room temperature for 20 min and then washed 3 × 5 min in pH 7.2 PBS buffer. The sections were permeabilized in pepsin digest all at 37°C for 40 min and washed 3 × 10 min in PBS. Thirdly, the sections were incubated in enzyme reaction mix (enzyme solution: label solution = 1 : 9 V) for 1 h at 37°C in a black wet box and then washed 3 × 10 min in PBS. At last, the nuclei were stained with 4,6-diamidino-2-phenylindole (DAPI, Sigma-Aldrich Co., St. Louis, MO, USA) at room temperature for 30 min. The sections were cover-slipped with glycerol-PBS (3 : 1 *v*/*v*) and examined by Leica 4000 at 488 nm and 350 nm (Leica, CA, Germany). The percentage of apoptosis was calculated using the following formula: Percentage of apoptosis(%) = numbers of apoptosis cells/total numbers of detected cells∗100%.

### 2.5. Immunofluorescence

The mice were anesthetized and transcardially perfused with 0.9% sterile saline, and after, the kidneys were prefixed with a little of 4% PFA/PBS, pH 7.2, then removed promptly and post-fixed in 4% PFA/PBS at 4°C for 4 h, and incubated successively in 15%, 20%, and 30% sucrose/pH 7.2 PBS solution to make them sink. At last, the kidneys were embedded in optimal cutting temperature (OCT) compound (Leica, CA, Germany). 12 *μ*m cryosections were collected by using Leica 3050 (Leica, CA, Germany) for immunofluorescence.

Immunofluorescence was performed as follows: after 1.5 h drying at 37°C, the sections were carried out for antigen retrieval in boiling 0.1 mol/l sodium citrate buffer for 15 min and incubated in pH 7.2 PBS buffer [including 0.3% Triton-100 or Tween-20 and 5% bovine serum albumin (BSA)] at 25°C for 1 h to block nonspecific binding site. Then, the sections were incubated overnight with rabbit antinuclear factor E2-related factor 2 (Nrf2, 1 : 500, ab62352) and rabbit antinicotinamide adenine dinucleotide phosphate oxidase 2 (NOX2, 1 : 500, ab80508), and related fluorescence secondary antibody was added for incubation of 1 h at 25°C. After DAPI was applied for 5 min, the sections were captured using Leica microscope.

### 2.6. Western Blot

The mice were deeply anaesthetized and sacrificed. The kidney tissues were immediately excised and homogenized in 1/5 (*w*/*v*) tissue protein extraction reagent (Thermo Fisher Scientific Inc., Waltham, MA, USA) and the protease inhibitor. The cold tissues were homogenized two times for 28 s with 30 s intervals using MM400 (Retsch GmbH, Haan, Germany) and centrifuged at 14000*g* for 30 min at 4°C to obtain the supernatants, and then, the supernatants were collected and stored at −86°C for Western blot analyses. The expression levels of apoptosome containing heme oxygenase 1 (HO-1), apoptosis protease-activating factor-1 (APAF-1), caspase-3, cleaved-caspase-3, B-cell lymphoma-2 (Bcl-2), Bcl-2-associated X protein (Bax), poly ADP ribose polymerase (PARP), cytochrome c, factor-associated suicide (FAS), Fas ligand (FASL), caspase-8, and NOX2 were assessed by Western blotting. Nrf2 levels in the cytoplasm and nuclear extracts of kidney tissues were assessed by Western blotting, which was obtained by a nuclear/cytoplasm fractionation kit (BioVision Inc., USA). Protein contents of the supernatants were measured by the bicinchoninic acid assay kit (Pierce Biotechnology Inc., Rockford, IL, USA).

Western blot analyses were performed following standard procedures. The supernatant proteins (30 *μ*g) were separated using sodium dodecyl sulfate polyacrylamide gel electrophoresis (SDS-PAGE, 120 V). Objective bands were transferred to a polyvinylidene difluoride membrane (PVDF; Roche Diagnostics Corporation, Basel, Switzerland) by 350 mA electrophoretic transfer. The membrane was blocked with 5% nonfat milk or 5% BSA in Tris-buffered saline (TBS, pH 7.2, containing 0.1% Tween-20) for 1 h at room temperature and incubated at 4°C overnight with the following primary antibodies, respectively: mouse anticytochrome c (15 KD, 31 KD, 45 KD, 1 : 1000, BD556432, Becton, Dickinson and Company Inc., Franklin Lakes, USA) and rabbit anti-Bax (22 KD, 1 : 1000, BD556467), rabbit anti-APAF-1 (130 KD, 1 : 1000, BD559683), rabbit anti-caspase-9 (37 KD, 39 KD, 49 KD, CST9504, Cell Signaling Technology Inc., Beverly, MA), rabbit anti-FASL (31 KD, ab15285), rabbit-caspase-12 (42 KD, 55 KD, CST2202), rabbit anti-FAS (48 KD, sc1032, Santa Cruz Biotechnology Inc., Dallas, TX, USA), mouse anti-*β*-actin (42 KD, 1 : 2000, Chemicon International Inc., California, USA), rabbit anti-Nrf2 (100 KD, ab62352), caspase-8 (18 KD, 43 KD, CST8592), rabbit anti-HO-1 (33 KD, ab68477), rabbit anti-PARP (55 KD, ab16572), and rabbit anti-NOX2 (65 KD, ab80508). Protein bands were detected using horseradish peroxidase- (HRP-) conjugated anti-rabbit or anti-mouse secondary antibodies (Cell Signaling Technology, Danvers, MA, USA). Protein bands were detected using FluorChem M system (Protein Simple, CA, USA). The mean optical density (OD) values of protein bands were measured with Scion image analysis software (Scion Corp., Frederick, MD, USA) and were normalized to mouse anti-*β*-actin as internal controls (OD detected protein/OD internal control).

### 2.7. Immunoprecipitation Assay

30 mg kidney tissues were homogenized in protein immunoprecipitation buffer including 1 × pH 7.2 PBS, 1% Triton X-100, and the protease inhibitor. The cold tissues were homogenized two times for 28 s with 30 s intervals using MM400 (Retsch GmbH, Haan, Germany) and centrifuged at 14000*g* for 30 min at 4°C to obtain the supernatants. After the supernatants were carried out to measure the protein content, 100 *μ*g protein supernatants were added 10 *μ*l of resuspended volume of protein A/G plus agarose and incubated for 30 min to wipe off the nonspecific adsorption. Then, the supernatants were added 2 *μ*g Nrf2 primary antibody (sc-30915) and incubated for 1 h at 4°C on a rotating device. And 20 *μ*l of resuspended volume of protein A/G plus agarose was added and incubated vibrationally overnight. The immunoprecipitates were collected by centrifugation at 1000*g* for 5 min at 4°C. The pellets were washed for 4 times with 1 ml immunoprecipitation buffer, each time repeating centrifugation step above. After the final wash, the pellets were resuspended in 40 *μ*l of 1× electrophoresis sample buffer. The samples were boiled for 3 min and analyzed 20 *μ*l aliquots by SDS-PAGE for ubiquitination (ab19247).

### 2.8. Statistical Analysis

All the data were analyzed by the software SPSS 15.0 (SPSS Software Inc., Chicago, IL, USA) statistically. Cell apoptosis rate, ROS level, GSH level, antioxidant enzyme activity, and Western blotting results were analyzed with Tukey's HSD post hoc test. Data were expressed as mean ± SEM and *p* < 0.05 was considered statistically significant.

## 3. Results

### 3.1. Troxerutin Inhibits Kidney Cell Apoptosis Induced by BDE-47 in the Mice

**Figure 1:**
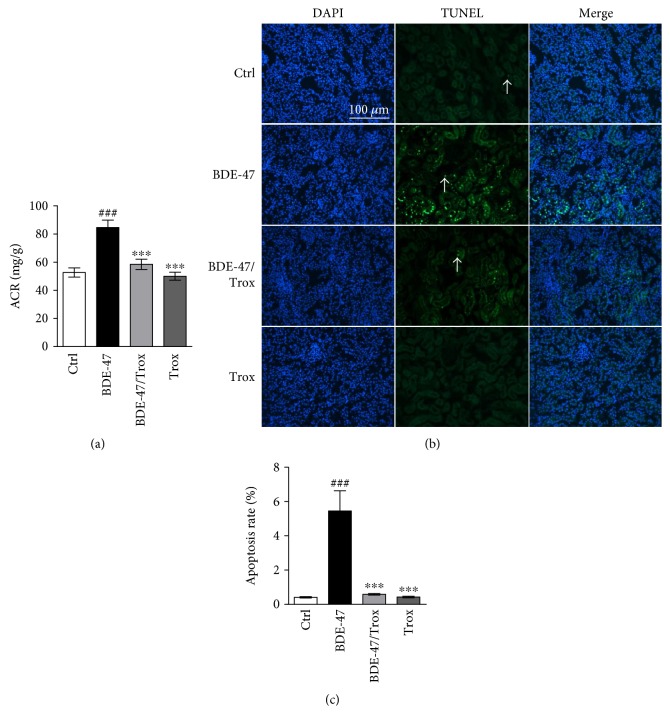
Troxerutin reduces ACR and kidney cell apoptosis in BDE-47 treated mice. (a) ACR (albumin-to-creatinine, mg/g, *n* = 8) was tested at 12 weeks after BDE-47 was orally treated. (b) Kidney cell apoptosis was examined by the use of TUNEL assay (20x). White arrows (green fluorescence) represent positive signals. 4′,6-Diamidino-2-phenylindole (DAPI) was used to stain the nuclei blue. Scale bar, 100 *μ*m. (c) TUNEL-positive cells were quantitatively analyzed in the kidney tissue by the use of Turkey's HSD post hoc test. Ctrl: control group; BDE-47: BDE-47-treated group; BDE-47 + Trox: BDE-47 and troxerutin cotreated group; Trox: troxerutin-treated group. Data were presented as mean ± SEM (*n* = 5). ^∗∗∗^*p* < 0.001 versus BDE-47-treated group. ^###^*p* < 0.001 versus control group.

Several tissues exhibit dysfunction caused by BDE-47, but it is unknown whether BDE-47 impairs the kidney in vivo. Therefore, we firstly used biochemical analysis and TUNEL assay to examine the change of kidney function and cell apoptosis of C57BL/6J mice treated with BDE-47. We found that BDE-47 treatment in mice significantly increased the ratio of albumin to creatinine (ACR, Figure 1(a)) and the number of kidney apoptosis cells compared to the untreated mice (Figures 1(b) and 1(c)). Orally administration of troxerutin in BDE-47-treated mice substantially reduced ACR and the ratio of kidney apoptosis cells compared to alone BDE-47-treated mice. However, there was no statistical difference among cotreated group with BDE-47 and troxerutin, troxerutin-treated group, and control group.

### 3.2. Troxerutin Ameliorates Kidney Mitochondrial Injury in BDE-47-Treated Mice

**Figure 2:**
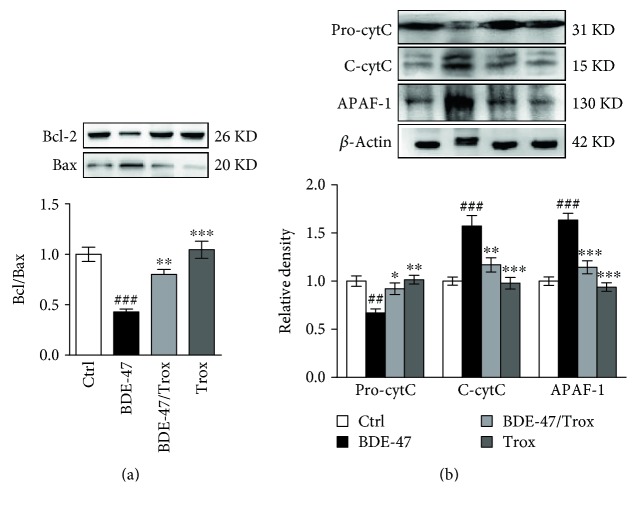
Troxerutin inhibited mitochondria abnormality caused by BDE-47. (a) Bcl-2 and Bax were measured by the use of Western blot analysis. (b) Cytochrome c and APAF-1 were detected by Western blot analysis. Pro-cytC means pro-cytochrome c in the mitochondria and c-cytC means cleaved cytochrome c in the cytosol. Data were showed as mean ± SEM (*n* = 5). ^∗^*p* < 0.05, ^∗∗^*p* < 0.01, and ^∗∗∗^*p* < 0.001 versus BDE-47-treated group. ^##^*p* < 0.01 and ^###^*p* < 0.001 versus control group.

Proapoptotic (Bax, Bad, etc.) and antiapoptotic (Bcl-2, Bcl-xL, etc.) proteins in Bcl-2 family regulate the process of apoptosis. We observed that BDE-47 treatment caused a markedly decline in Bcl-2 expression and an increase in Bax expression; however, troxerutin supplementation restored the ratio of Bcl-2 to Bax (Figure 2(a)). The reduction of Bcl-2/Bax ratio triggered cytochrome c transferring from the mitochondria to the cytoplasm. Additionally, BDE-47 reduced the number of mitochondrial cytochrome c fraction and elevated the number of cytosolic cytochrome c fraction (Figure 2(b)). However, troxerutin supplementation reversed the change and restored the location of cytochrome c; at the same time, we found that troxerutin inhibited the increase of APAF-1 protein induced by BDE-47 (Figure 2(b)), but troxerutin treatment alone did not alter these parameters (Figures 2(a) and 2(b)).

### 3.3. Troxerutin Blocks the Activities of Kidney Caspase Proteins in BDE-47-Treated Mice

**Figure 3:**
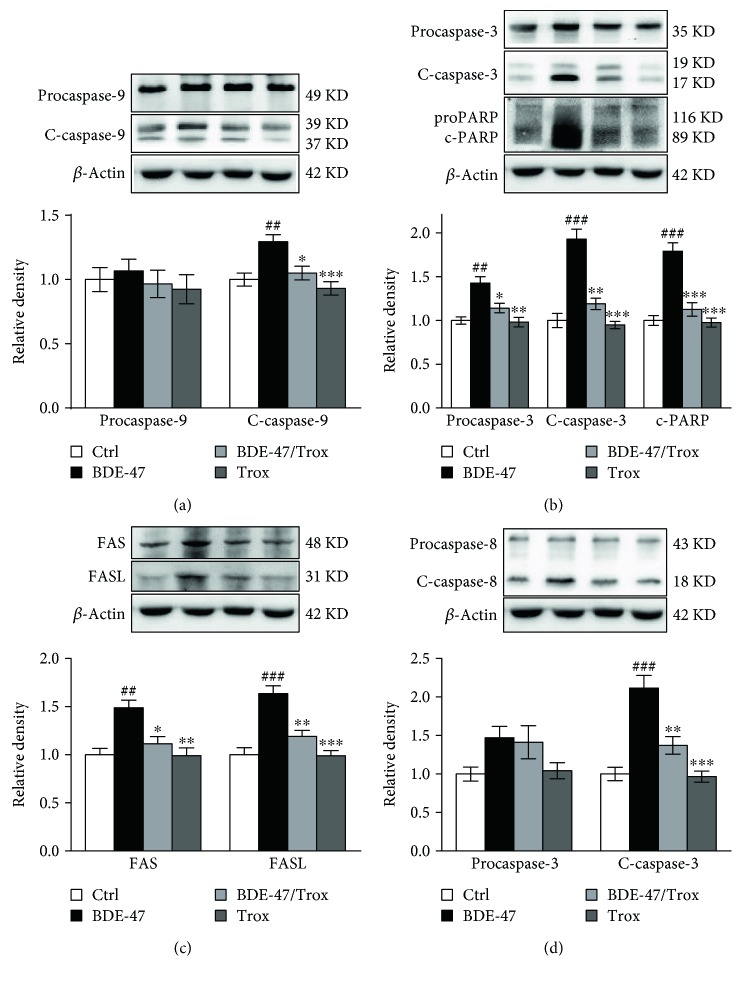
Troxerutin antagonizes BDE-47 induced expression of kidney apoptosis molecules. (a) Procaspase-9 and cleaved caspase-9 (c-caspase-9) were examined by Western blot analysis. (b) Procaspase-3, cleaved caspase-3 (c-caspase-3), proPARP, and cleaved PARP (c-PARP) were detected by Western blot. (c) FAS and FASL were detected by Western blot. (d) Procaspase-8 and cleaved caspase-8 (c-caspase-8) were measured by Western blot. Data were expressed as mean ± SEM (*n* = 5). ^∗^*p* < 0.05, ^∗∗^*p* < 0.01, and ^∗∗∗^*p* < 0.001 versus BDE-47-treated group. ^##^*p* < 0.01 and ^###^*p* < 0.001 versus control group.

The release of cytochrome c from the mitochondria to the cytoplasm promotes the forming of apoptosome containing APAF-1, cytochrome c, and procaspase-9; then, procaspase-9 is turned into caspase-9 to activate the downstream executioners caspase-3, caspase-6, and caspase-7, resulting in PARP cleavage, and initiates cell apoptosis. We found that BDE-47 had no significant effect on procaspase-9 level (Figure 3(a)) and remarkably increased procaspase-3 level (Figure 3(b)), enhanced the levels of cleaved caspase-9 and cleaved caspase-3, and consequently promoted the cleavage of PARP (Figure 3(b)). We observed the increase of FAS, FASL, and cleaved caspase-8 in the kidney of BDE-47-treated mice, while there was no significant change of procaspase-8 expression compared to control group (Figures 3(c) and 3(d)). However, troxerutin inhibited the activation of caspase-9 and caspase-3 and reduced the level of cleaved PARP, FAS, FASL, and cleaved caspase-8. There was no significant difference among control group, BDE-47 and troxerutin cotreated group, and troxerutin group.

### 3.4. Troxerutin Increases Kidney Antioxidant Capacity in BDE-47-Treated Mice

**Figure 4:**
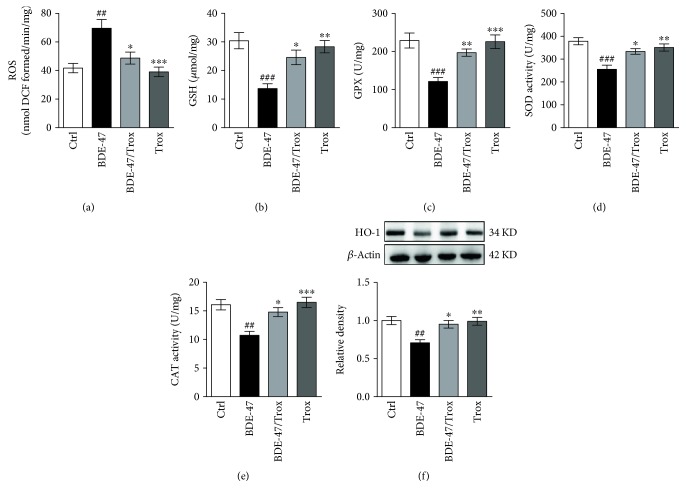
Troxerutin increases kidney antioxidative level in BDE-47-treated mice. (a) ROS level was examined by fluorescent probe DCFH-DA. (b–e): GSH content (b), GPx activity (c), SOD activity (d), and CAT activity (e) were measured by the use of biochemical assay. (f) HO-1 activity was assessed by Western blot analysis. Data were expressed as mean ± SEM (*n* = 5). ^∗^*p* < 0.05, ^∗∗^*p* < 0.01, and ^∗∗∗^*p* < 0.001 versus BDE-47-treated group. ^##^*p* < 0.01 and ^###^*p* < 0.001 versus control group.

Increasing ROS formation plays a key role in the tissue damage induced by BDE-47. To investigate whether troxerutin inhibits oxidative stress, we observed the levels of ROS and glutathione (GSH) and the activities of antioxidative enzymes. The results showed BDE-47 treatment led to a pronounced increase of ROS generation (Figure 4(a)) and a decrease of GSH content (Figure 4(b)) in the mouse kidney while troxerutin administration reduced ROS content and enhanced GSH level, then protected the mouse kidney against oxidative insult. Additionally, we found that BDE-47 significantly alleviated the activities of GPx (Figure 4(c)), SOD (Figure 4(d)), CAT (Figure 4(e)), and HO-1 (Figure 4(f)) in the kidney of mice compared to control mice, and troxerutin administration inhibited the reduction of the activities of these antioxidant enzymes.

### 3.5. Troxerutin Enhances Kidney Nrf2 Activity in BDE-47-Treated Mice

**Figure 5:**
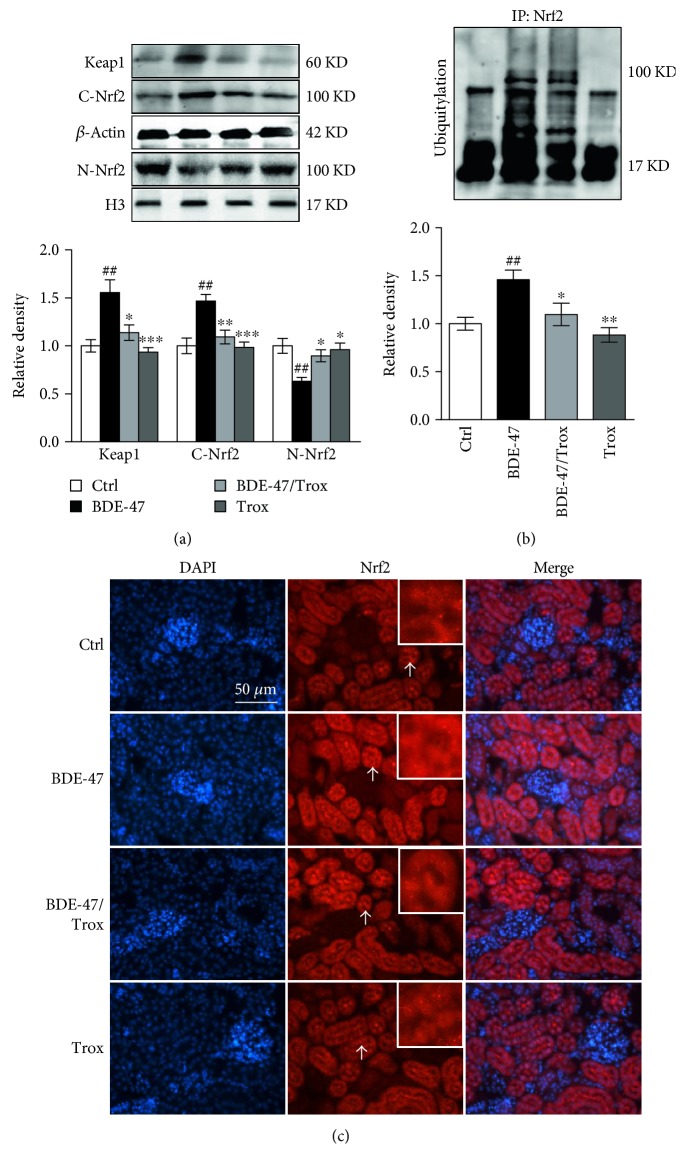
Troxerutin increases kidney Nrf2 activity in BDE-47-treated mice. (a) The activities of Keap1, cytoplasmic Nrf2, and nuclear Nrf2 were detected by Western blot assay. (b) Nrf2 ubiquitylation was evaluated by Western blot analysis after Nrf2 immunoprecipitation. (c) Nrf2 immunohistochemical staining. Green fluorescence represents positive signal, and DAPI was used to stain the nuclei blue. Scale bar, 50 *μ*m. White arrow indicates positive signal which was placed in the upper right corner. Data were expressed as mean ± SEM (*n* = 5). ^∗^*p* < 0.05, ^∗∗^*p* < 0.01, and ^∗∗∗^*p* < 0.001 versus BDE-47-treated group. ^##^*p* < 0.01 versus control group.

Nrf2, a major ROS-regulating effector, induces the increase of many antioxidative genes, such as SOD, CAT, GPx, and HO-1, via binding to their promoters [28]. Then, we investigated the status of the intracellular antioxidant defense mechanisms by detecting the activity of Nrf2. The results showed that BDE-47 administration significantly increased the expression of Nrf2-binding protein Keap1 (Kelch-like ECH-associated protein 1) in the kidney tissue of mice compared to control group (Figure 5(a)), resulting in the increase of Nrf2 ubiquitylation (Figure 5(b)) and in the reduction of nuclear Nrf2 activity (Figure 5(a)). However, troxerutin supplementation decreased the ubiquitination of Nrf2 and increased the activity of nuclear Nrf2 in the kidney of BDE-47-treated mice. Immunofluorescence staining showed a strong expression signal of Nrf2 presented in the cytoplasm; however, there was a weak signal in the nuclear after treating mice with BDE-47, and furthermore, troxerutin blocked this abnormal change (Figure 5(c)). The results suggest that troxerutin enhanced the activity of Nrf2 and prevented the kidney damage induced by BDE-47.

### 3.6. Troxerutin Inhibits Kidney NOX2 Expression in BDE-47-Treated Mice

**Figure 6:**
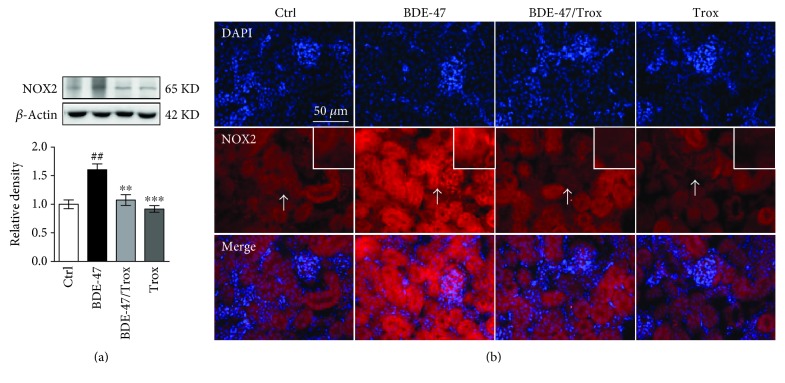
Troxerutin decreases the activity of kidney NOX2 in BDE-47-treated mice. (a) NOX2 activity was evaluated by Western blot analysis. (b) NOX2 immunohistochemical staining. Green fluorescence represents positive signal, and DAPI was used to stain the nuclei blue. Scale bar, 50 *μ*m. White arrows indicate positive signals which were placed in the upper right corner. Data were expressed as mean ± SEM (*n* = 5). ^∗∗^*p* < 0.01 and ^∗∗∗^*p* < 0.001 versus BDE-47-treated group. ^##^*p* < 0.01 versus control group.

NOX2, one of NADPH oxidases, is richly expressed in kidney tubular cells and endothelial cells and identified as a major source of oxidative stress in renal disease progression [29]. The results showed that BDE-47 supplement markedly raised the activity of NOX2, which was reversed by troxerutin (Figure 6(a)). There was no significant difference among control, cotreated group with BDE-47/troxerutin, and troxerutin group.  Figure 6(b) also shows that there was a strong fluorescence signal in kidney cell membrane in BDE-47-treated mice, while troxerutin treatment significantly attenuated fluorescence signal of NOX2 induced by BDE-47.

## 4. Discussion

PBDEs are widely present in the household and environment and have attracted people's attention because of their toxic action on animals including neurotoxicity, reproductive toxicity, hepatotoxicity, and endocrine toxicity. PBDEs have been detected in the blood, lipid tissue, and breast milk of human [30–32], which makes up a potential health risk. The investigations show that PBDEs significantly affect infant birth weight and birth length, thyroid function in young children, and neurodevelopment [33, 34]. Furthermore, the toxicology of PBDEs is intimately associated with hydroxylated products of PBDEs [34, 35]. Among PBDE congeners, BDE-47 has the highest concentration and the strongest toxicity in the environment. Oxidative stress has been demonstrated to play an important role in BDE-47's toxic action. Increasing data reveal that BDE-47 induces oxidative stress and in turn mediates DNA damage, mitochondrial dysfunction, and endoplasmic reticulum stress [16, 36, 37]. Recently, antioxidants have been demonstrated to eliminate oxidative stress by scavenging ROS induced by BDE-47 in vitro [13, 14, 38]. Interestingly, we find that troxerutin could effectively reduce oxidative stress-mediated NAD^+^ depletion and ameliorate liver inflammation injury. In this present study, we further found that troxerutin decreases renal cell apoptosis caused by BDE-47 via enhancing Nrf2 activity and blocking NOX2 activity in mice.

Oxidative stress is considered as a disorder between oxidant production and antioxidant defense system. The NADPH oxidase is a major ROS-generating enzyme, which has seven isoforms including NOX1–NOX5, Duox1, and Duox2. NOX2 (isoform 2, gp91phox), constitutively expressed in kidney tubular cells and endothelial cells, is identified as a major source of oxidative stress in kidney diseases [29]. BDE-47 accumulation can result in metabolic disorders such as the disruption of glycolipid metabolism, the reduction of testosterone, and the disruption of the seminiferous epithelium [39, 40], Herein, we observed that BDE-47 increased renal NOX2 expression and caused ROS accumulation in the kidney tissue of mice and might be associated with metabolic disorders [41], while troxerutin reversed the increase of NOX2 activity and ROS level, which is in line with flavonoid purple sweet potato color reducing the NOX2 activity by interrupting the assembly of catalytic subunit gp91phox and regulatory subunits in the brain of domoic acid-treated mice [42].

In vivo, there is a dynamic equilibrium between generation and elimination of free radical. NOX2 is a major ROS-generating enzyme, whereas Nrf2 is an important antioxidative transcription factor, which is bound with its inhibitor Keap1 in the cytoplasm under resting condition, where Keap1 promotes the ubiquitination and proteasome degradation of Nrf2 via E3 ligase system [43]. Upon exposure to stress or to chemical inducers, Nrf2 is freed from Keap1, translocating to the nucleus and inducing the transcription of downstream genes including HO-1, SOD, GPx, and CAT which play vital roles in antioxidative response. The Keap1-Nrf2 system is thought to be a crucial role in kidney oxidative injury and considered as a prospective target for kidney disease [43, 44], so proteasome inhibitor MG132 could inhibit Nrf2 proteasomal degradation and promote antioxidative activity, having the protective efficacy on diabetes nephropathy [45]. BDE-47 induced oxidative damage and inflammatory response in a human extravillous trophoblast cell line, HTR-8/SVneo, while pretreatment with tert-butyl hydroquinone or sulforaphane, known Nrf2 inducers, reduced the nuclear import of redox-sensitive transcription factor nuclear factor kappa B (NF-*κ*B) and release of inflammatory factor interleukin 6 (IL-6) [46]. Previous reports demonstrate that Keap1 is induced under diabetic nephropathy [47] and then whether BDE-47 as a diabetic prevalence factor regulates the level of Keap1 [39]. Here, we found that long-term treatment of BDE-47 increased Keap1 level and made Nrf2 ubiquitination degradation, leading to the activity reduction of Nrf2 and its downstream genes including CAT, GPx, SOD, and HO-1 in the kidney of mice. However, troxerutin partly mimicked the effect of MG132, blocked the ill effect of BDE-47, and increased the Nrf2 activity, which is consistent with the previous report, in which polyphenol blunts the Nrf2 transcriptional depression [48].

The increase of NOX2 activity and the decrease of Nrf2 in the tissue cause harmful oxidative stress and then initiate apoptotic event. Apoptosis is mediated through intrinsic pathways and extrinsic pathways [49]. Oxidative stress mediates intrinsic apoptosis pathway and triggers event upstream of mitochondrial apoptosis in kidney tissue cells [50, 51]. Bcl-2 family proteins are involved in intrinsic apoptosis pathway; therefore, the downregulation of Bcl-2/Bax ratio promotes the release of cytochrome c from the mitochondria to the cytoplasm under oxidative stress and then induces the forming of an apoptosome containing APAF-1, cytochrome c, and procaspase-9. Procaspase-9 is processed to cleaved caspase-9 via an intrinsic autocatalytic activity of itself, which allows caspase-9 cleavage to activate the downstream executioners including caspase-3, caspase-6, and caspase-7 and causes the cleavage of PARP and subsequent apoptosis. The extracellular pathways are also activated under stresses and promote the apoptosis protein expression of FAS, TNF*α*, and caspase-8, further activate caspase-3, and trigger cell apoptosis. The previous data showed that caspase-3 was significantly activated in the liver and cerebellum of mice after exposure to BDE-47 [52]. BDE-47 also increased the gene expression level of caspase-3, caspase-8, caspase-9, TNFR1, and Bax and impaired macrophage accessory cell function in a concentration-dependent manner [53]. We have demonstrated that BDE-47 could increase caspase-3 activity and Bax levels and decline Bcl-2 level in the liver of mice [16]. In this study, BDE-47 is further demonstrated to be able to elevate the ratio of Bcl-2/Bax, promote the release of cytochrome c from the mitochondria to the cytoplasm, and increase the expression of apoptosis proteins including APAF-1, caspase-9, caspase-3, and PARP, resulting in triggering intracellular apoptosis pathway. In addition, we also find that BDE-47 increases the protein expression of FAS, FASL, and caspase-8, activating the extracellular apoptosis pathway. However, the treatment with troxerutin deletes the adverse effect caused by BDE-47 and blocks the number of TUNEL-positive cells in the kidney tissue of mice, which is similar to the result of troxerutin alleviating oxidative damage and kidney cell apoptosis induced by D-galactose [54].

In summary, BDE-47 treatment for a long time enhances the ubiquitination of Nrf2, lessens the activity of Nrf2 and its downstream antioxidative enzymes including SOD, GPx, and CAT, and promotes oxidative stress of kidney cells of mice, increasing mitochondrial cytochrome c into the cytoplasm, triggering the mitochondrial apoptosis pathway. On the other hand, BDE-47 increases the expression of FAS, FASL, and caspase-8, starting up an extrinsic pathway. Furthermore, troxerutin inhibits these disadvantages induced by BDE-47 and decreases intrinsic and extrinsic apoptosis pathway, protecting kidney cells from oxidative stress-induced apoptosis damage.

## Supplementary Material

Figure S1. Different doses of BDE-47 increased ACR production and kidney ROS accumulation in the mice. (A) The results of ACR ratio (Urine albumin-to-creatinine) after BDE-47 were given by gavage for 12 weeks (n=8). (B) Kidney ROS content was detected by fluorescent probe 2′, 7′-dichlorofluorescin diacetate (DCFH-DA) after BDE-47 were given by gavage for 12 weeks (n=5). ∗P<0.05 and ∗∗∗P<0.001 versus control (Ctrl) group. The data were analyzed with One-way ANOVA followed by post hoc Tukey test. Figure S2. Different doses of Troxerutin reduced ACR production and kidney ROS content induced by BDE-47 in the mice. (A) The results of ACR ratio after BDE-47 (50mg/kg/day) and Troxerutin were administrated by gavage for 12 weeks (n=8). (B) Kidney ROS level was detected DCFH-DA after BDE-47 and Troxerutin were orally given for 12 weeks (n=5). ∗P<0.05, ∗P<0.01 and ∗∗∗P<0.001 versus BDE-47 group. The data were analyzed with One-way ANOVA followed by post hoc Tukey test.

## Abbreviations

ROS: Reactive oxygen species
DCFH-DA: Dichlorofluorescein diacetate
BDE-47: 2,2′,4,4′-Tetrabromodiphenyl ether
PBDEs: Polybrominated diphenyl ethers
POPs: Persistent organic pollutants
T3: Triiodothyronine
T4: Tetraiodothyronine
GSH: Glutathione
SOD: Superoxide dismutase
CAT: Catalase
GPx: Glutathione peroxidase
TUNEL: Terminal deoxynucleotidyl transferase-mediated dUTP nick end labeling
APAF-1: Apoptosome containing apoptosis protease-activating factor-1
PARP: Poly ADP ribose polymerase
HO-1: Heme oxygenase-1
Bcl-2: B-cell lymphoma-2
Bax: Bcl-2-associated X protein
FAS: Factor-associated suicide
FASL: Fas ligand
Nrf2: Nuclear factor E2-related factor 2
NOX2: Nicotinamide adenine dinucleotide phosphate oxidase 2
PBS: Phosphate buffer saline solution
DAPI: 4,6-Diamidino-2-phenylindole
SDS-PAGE: Sodium dodecyl sulfate polyacrylamide gel electrophoresis
PVDF: Polyvinylidenedifluoride membrane
BSA: Bovine serum albumin
TBS: Tris-buffered saline
NF-*κ*B: Nuclear factor kappa B
IL-6: Interleukin 6.
