# Supplementary material for: Troxerutin Reduces Kidney Damage against BDE-47-Induced Apoptosis via Inhibiting NOX2 Activity and Increasing Nrf2 Activity
Source: Oxid Med Cell Longev. 2017 Oct 15;2017:6034692. doi: 10.1155/2017/6034692 (PMC5661100; doi:10.1155/2017/6034692)
Supplement: Supplementary file 1 — Figure S1. Different doses of BDE-47 increased ACR production and kidney ROS accumulation in the mice. (A) The results of ACR ratio (Urine albumin-to-creatinine) after BDE-47 were given by gavage for 12 weeks (n=8). (B) Kidney ROS content was detected by fluorescent probe 2′, 7′-dichlorofluorescin diacetate (DCFH-DA) after BDE-47 were given by gavage for 12 weeks (n=5). ∗P<0.05 and ∗∗∗P<0.001 versus control (Ctrl) group. The data were analyzed with One-way ANOVA followed by post hoc Tukey test. Figure S2. Different doses of Troxerutin reduced ACR production and kidney ROS content induced by BDE-47 in the mice. (A) The results of ACR ratio after BDE-47 (50mg/kg/day) and Troxerutin were administrated by gavage for 12 weeks (n=8). (B) Kidney ROS level was detected DCFH-DA after BDE-47 and Troxerutin were orally given for 12 weeks (n=5). ∗P<0.05, ∗P<0.01 and ∗∗∗P<0.001 versus BDE-47 group. The data were analyzed with One-way ANOVA followed by post hoc Tukey test. [file 6034692.f1.docx]

**Figure S1.** Different doses of BDE-47 increased ACR production and kidney ROS accumulation in the mice. (A) The results of ACR ratio (Urine albumin-to-creatinine) after BDE-47 were given by gavage for 12 weeks (n=8). (B) Kidney ROS content was detected by fluorescent probe 2′, 7′-dichlorofluorescin diacetate (DCFH-DA) after BDE-47 were given by gavage for 12 weeks (n = 5). *P < 0.05 and ***P < 0.001 versus control (Ctrl) group. The data were analyzed with One-way ANOVA followed by post hoc Tukey test.





Figure S2. Different doses of Troxerutin reduced ACR production and kidney ROS content induced by BDE-47 in the mice. (A) The results of ACR ratio after BDE-47 (50mg/kg/day) and Troxerutin were administrated by gavage for 12 weeks (n=8). (B) Kidney ROS level was detected DCFH-DA after BDE-47 and Troxerutin were orally given for 12 weeks (n= 5). *P < 0.05, *P<0.01 and ***P < 0.001 versus BDE-47 group. The data were analyzed with One-way ANOVA followed by post hoc Tukey test.
