# Supplementary figures and images for: Troxerutin Reduces Kidney Damage against BDE-47-Induced Apoptosis via Inhibiting NOX2 Activity and Increasing Nrf2 Activity
Source: Oxid Med Cell Longev. 2017 Oct 15;2017:6034692. doi: 10.1155/2017/6034692 (PMC5661100; doi:10.1155/2017/6034692)

**A**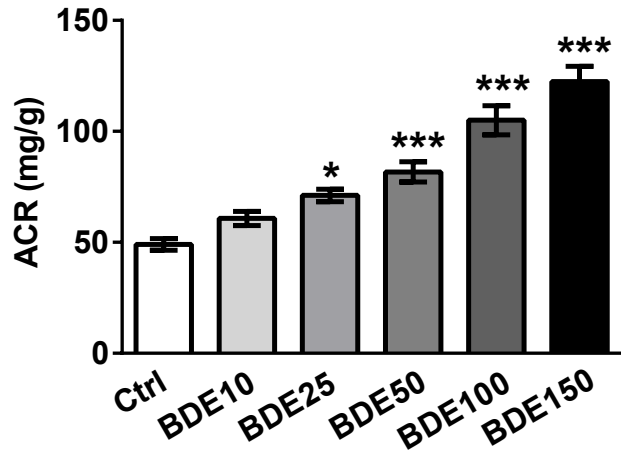**B**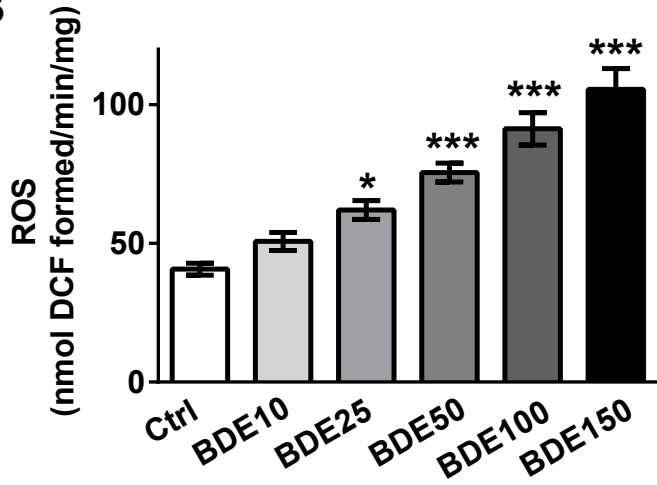

Supplement: Supplementary file 2 [file 6034692.f2.pdf]

**A**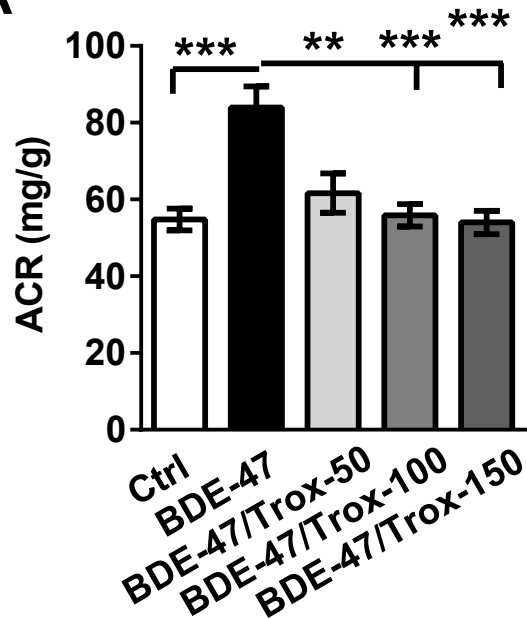**B**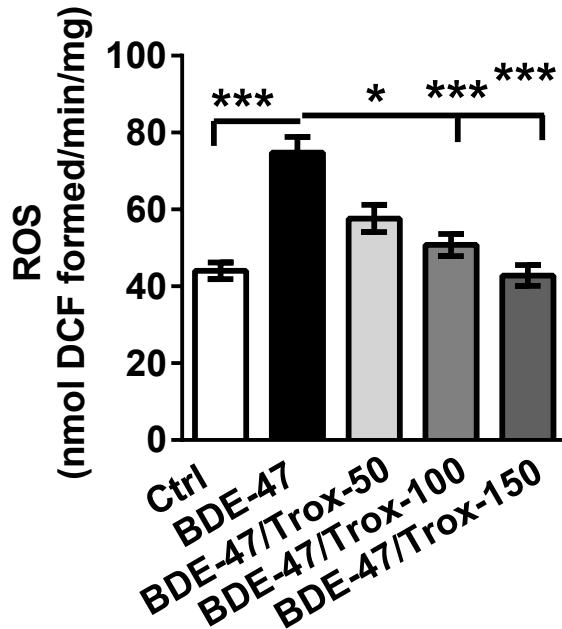

Supplement: Supplementary file 3 [file 6034692.f3.pdf]
